# Frontal fibrosing alopecia in association with Sjögren's syndrome: more than a simple coincidence*

**DOI:** 10.1590/abd1806-4841.20164526

**Published:** 2016

**Authors:** Karina Colossi Furlan, Priscila Kakizaki, Juliana Cabral Nunes Chartuni, Neusa Yuriko Sakai Valente

**Affiliations:** 1 Hospital do Servidor Público Estadual do Estado de São Paulo (HSPE) - São Paulo (SP), Brazil; 2 Hospital Regional de Santa Maria - Brasília (DF), Brazil; 3 Universidade de São Paulo (USP) - São Paulo (SP), Brazil

**Keywords:** Alopecia, Autoimmunity, Discoid lupus erythematosus, Lichen planus, Sjögren's Syndrome

## Abstract

Frontal fibrosing alopecia is a distinctive form of scarring alopecia considered to be a clinical variant of lichen planopilaris. It predominantly occurs in postmenopausal women and has a slowly progressive course. It was first described by Kossard in 1994. Since then the number of reported cases has increased significantly. Coexistence of frontal fibrosing alopecia and autoimmune disorders - such as discoid erythematosus lupus and Sjögren's syndrome - may suggest a common pathogenic background among the diseases.

## INTRODUCTION

Frontal fibrosing alopecia (FFA) is a scarring alopecia considered a clinical variant of lichen planopilaris (LPP).^1^ Although more common in postmenopausal women, the disease may also affect premenopausal women. The condition is clinically characterized by a symmetrical frontotemporal hairline recession (marginal alopecia).^1^ It rarely occurs in males, with only one case of beard involvement reported.^2^ Familiar involvement has also been suggested.^1^ The affected skin is pale and atrophic. Perifollicular papules can be observed in approximately 60% of cases and follicular hyperkeratosis, female androgenetic alopecia, pruritus, and lichen planus in 30% of cases.^1^ There is a contrast in color between the uniformly pale alopecia area and the sun-damaged skin of the forehead.^1^ Bilateral loss of eyebrow is a common finding, which can occur before or after the frontal hair loss.^1^ Some patients present with hair loss on the forearms, armpits, and upper and lower limbs.^1^

Mulinari-Brenner et al. (2007) reported 6 cases of FFA, which were the first cases reported in Brazil. The authors observed that all the patients were postmenopausal women, with hair rarefaction evolution ranging from 1½-10 years and a mean age of 65.8 years (54-82).^3^

Clinical examination reveals hyperkeratosis and erythematous-violaceous coloration in some follicular orifices, which is an early sign that can be observed with magnifying glasses.^1^ Serum levels of androgens, thyroid hormones, and hematological indices are normal. Anti-DNA and FAN can be positive at low titers.^1^ Histological examination of the affected scalp reveals scarring alopecia with mononuclear lichenoid infiltrate involving the infundibulum and isthmus of the folicle.^1^ Direct immunofluorescence is negative. Possible diagnoses to be ruled out include alopecia areata, cutaneous and systemic lupus, traction alopecia, androgenetic alopecia, pseudopelade of Brocq, and familiar high frontal hairline.^3^

There is a growing number of FFA reports in association with autoimmune diseases, in particular with discoid lupus erythematosus (DLE).^4-6^ DLE shares the interface dermatitis pattern with inflammatory T-cell infiltrate, which may suggest a common autoimmune etiology for both diseases.^5^ We present the second report of association between AFF and Sjögren's syndrome (SS).^7^

## CASE REPORT

**Figure 1:**
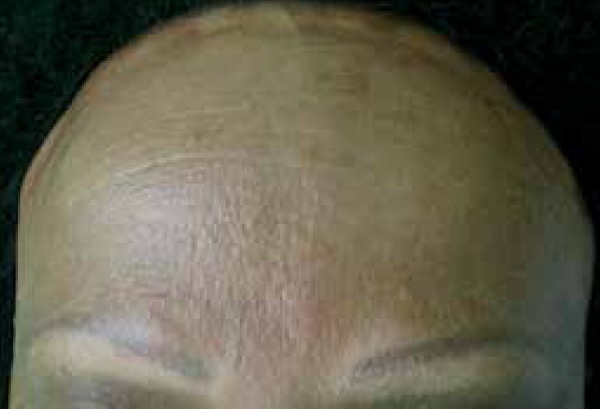
Marginal frontoparietal alopecia and absence of eyebrows

**Figure 2:**
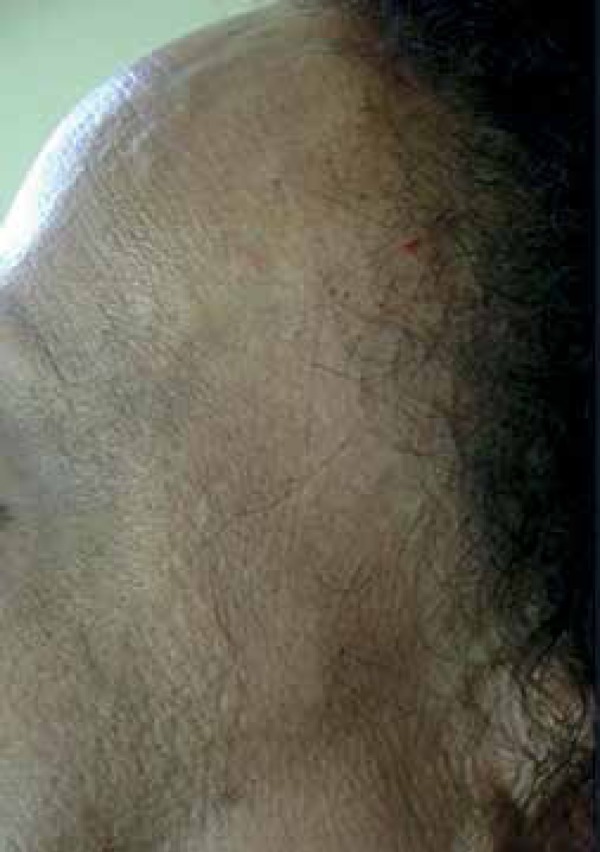
Left marginal alopecia with lonely hair

**Figure 3:**
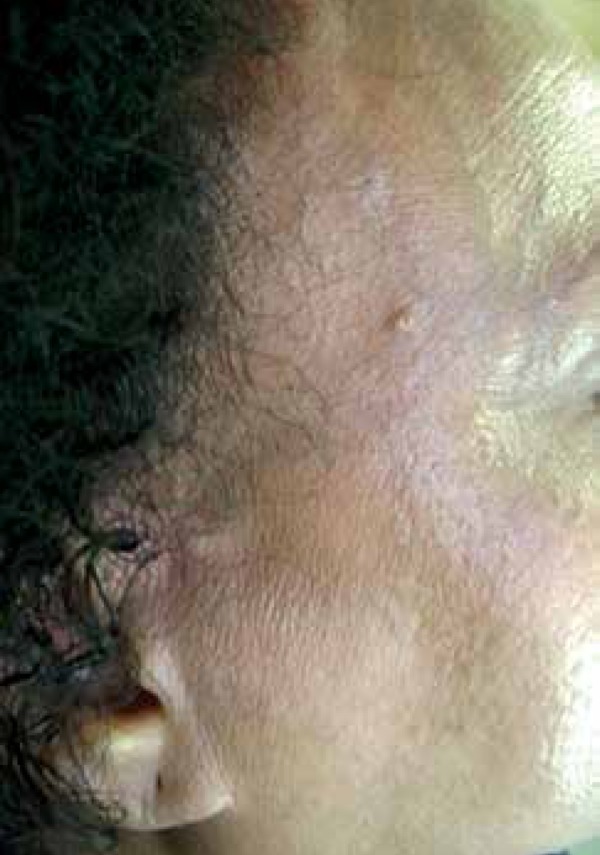
Right marginal alopecia with lonely hairs

**Figure 4:**
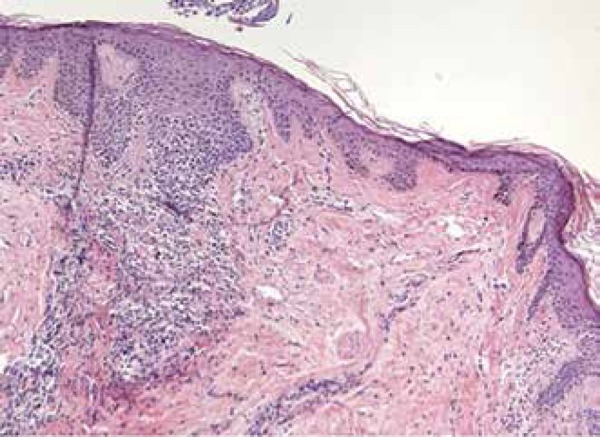
Lichenoid perifolliculitis and scarring in the dermis (Hematoxylin - eosin, x100)

**Figure 5:**
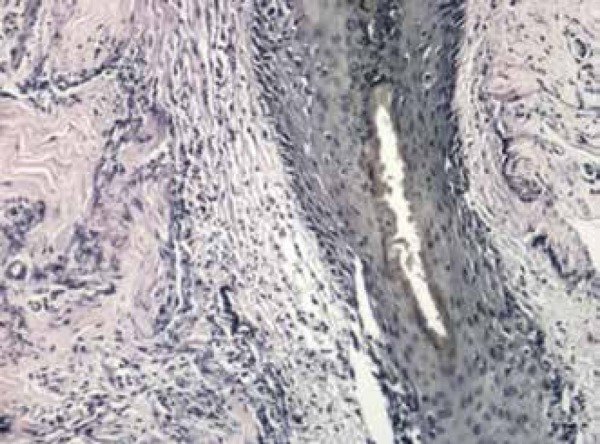
Partial destruction of elastic perifollicular fibers (Verhoeff) (Hematoxylin - eosin, x200)

**Figure 6:**
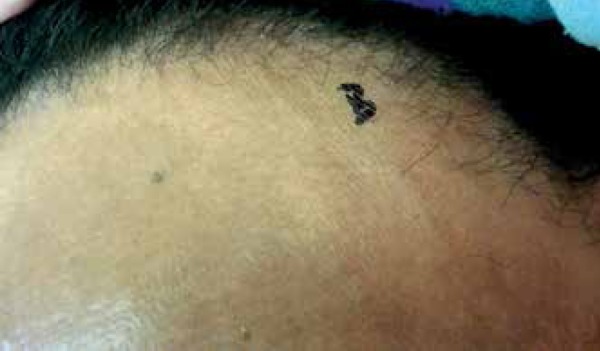
Detail of the left frontoparietal implantation line. Perifollicular papules on the alopecia affected area

We report a 50-year-old female menopausal black patient presented with progressive hair loss in the frontotemporal and eyebrow regions for one year. She reports oral mucosa and eye dryness with 10 years of evolution. Clinical examination revealed bilateral patches of scarring alopecia in the frontotemporal region and absence of eyebrows (Figures 1-3). Alopecia areas in the frontoparietal region reveal perifollicular papules (Figure 4). The patient tested positive for ANA (1/80). Histopathological examination of the right frontal hair implantation line revealed lichenoid perifolliculitis and scarring in the dermis, consistent with lichen planopilaris, in correlation with the clinical examination that suggested FFA (Figure 5). Immunohistochemistry analysis revealed partial destruction of elastic perifollicular fibers (Figure 6). Schirmer test was also positive. Histopathological examination of the salivary gland revealed duct ectasia, edema, and local lymphocytic infiltrate, compatible with Sjögren's syndrome.

Treatment was performed with intradermal injections of triamcinolone (40mg/mL, 1:10 dilution) in the frontotemporal region with monthly finasteride 2mg/day PO, chloroquine 250mg/day PO, and minoxidil 5% topical daily. After 4 years of regular follow up, we observed stabilization with no regrowth of in scarred areas.

## DISCUSSION

The etiology of FFA remains unknown. FFA and LPP share these microscopic features: lymphocytic inflammatory infiltrate around the isthmus and infundibulum of the hair follicles; presence of apoptotic cells in the outer root sheath; and perifollicular concentric fibrosis, which result in scarring alopecia.^1^ Specific histopathological findings reveal greater degree of apoptosis and reduced inflammation in FFA in relation to LPP.^6^ Clinically, FFA occurs more often after menopause and in the frontotemporal region. These features contrast with classical multifocal areas of scarring alopecia in LPP.^6^ Studies also describe the association of the HLA-DR1 (DR1 subtype of human leukocyte antigen) system with LPP. HLA is part of the major histocompatibility complex class II and is a ligand for T lymphocytes.^8^ However, cutaneous lesions and mucosal lichen planus have been described in patients with FFA.^6^

Without therapeutic interference, the progression of the condition is common. The frontal recession of the hairline can progress up to half of the scalp or more, but the progression of the lesion is variable.^6^ Although the disease seems to be self-limiting in most cases, the degree of progression before stabilization is unpredictable.^1^ However, the reversal of the fibrosis has never been described. Suggested treatments include: topical, intralesional, and systemic corticosteroids; topical retinoids; oral isotretinoin ; topical minoxidil ; hydroxychloroquine; and finasteride. Oral finasteride (2.5mg daily) combined with minoxidil 2% halted the progression of alopecia in some patients after 12-18 months of treatment.^9^ Some authors suggest that the combination of oral dutasteride with topical calcineurin inhibitor can represent a safe and effective alterna tive therapy for AFF.^9^

Gaffney et al. reported a case of discoid lupus erythematosus complicated by FFA in a 69-year-old patient. Alopecia developed after two years of evolution of the DLE with manifestations on the scalp, face, neck and thorax.^4^ Khan et al. reported the case of a 46-year-old patient with clinical and histopathological diagnosis of FFA that later showed photosensitivity at hair loss sites. A biopsy confirmed the diagnosis of AFF, but direct immunofluorescence was positive for IgM, IgG, IgA, and fibrin along the basement membrane zone, consistent with the diagnosis of lupus erythematosus.^10^ Shapiro et al. reviewed 62 cases and identified 18% of patients with autoimmune disorders of connective tissue in association with FFA, which are: systemic lupus erythematosus, DLE, rheumatoid arthritis, and polymyositis.^6^ Tosti et al. have also recently reported 7 cases of clinical coexistence of FFA and DLE on the scalp.^5^ In 2008 Takahashi *et al.* described the first and only case of association between AFF and SS in a postmenopausal Asian patient.^7^

Reports of DLE and FFA association, as well as association with other autoimmune conditions, suggest the evidence of an autoimmune etiology for this condition.^5^ Clearly, FFA is still a little known condition with few effective treatment options available. The present case report complements the dermatologic literature that highlights the importance of research of other autoimmune diseases associated with FFA given the therapeutic and prognostic implications of these associations.
